# Clinical outcomes of scalp or face angiosarcoma treatment with intensity-modulated radiotherapy: a multicenter study

**DOI:** 10.1093/jrr/rrad089

**Published:** 2023-11-22

**Authors:** Takahiro Iwai, Toshiyuki Imagumbai, Shinya Hiraoka, Takahiro Kishi, Shun Okabayashi, Ryo Ashida, Takamasa Mitsuyoshi, Yukinori Matsuo, Takashi Ishigaki, Takashi Mizowaki, Masaki Kokubo

**Affiliations:** Department of Radiation Oncology, Kobe City Medical Center General Hospital, Minamimachi 21-1, Minatojima, Chuo-ku, Kobe 650-0047, Hyogo, Japan; Department of Radiation Oncology and Image-Applied Therapy, Graduate School of Medicine, Kyoto University, 54 Shogoin-Kawahara-cho, Sakyo-ku, 606-8507, Kyoto, Japan; Department of Radiation Oncology, Kobe City Medical Center General Hospital, Minamimachi 21-1, Minatojima, Chuo-ku, Kobe 650-0047, Hyogo, Japan; Department of Radiation Oncology and Image-Applied Therapy, Graduate School of Medicine, Kyoto University, 54 Shogoin-Kawahara-cho, Sakyo-ku, 606-8507, Kyoto, Japan; Department of Radiation Oncology, Osaka Red Cross Hospital, 5-30 Fudegasaki-cho, Tennoji-ku, 543-8555, Osaka, Japan; Department of Radiation Oncology, Kobe City Medical Center General Hospital, Minamimachi 21-1, Minatojima, Chuo-ku, Kobe 650-0047, Hyogo, Japan; Department of Radiation Oncology, Kobe City Medical Center General Hospital, Minamimachi 21-1, Minatojima, Chuo-ku, Kobe 650-0047, Hyogo, Japan; Department of Radiation Oncology, Kobe City Medical Center General Hospital, Minamimachi 21-1, Minatojima, Chuo-ku, Kobe 650-0047, Hyogo, Japan; Department of Radiation Oncology and Image-Applied Therapy, Graduate School of Medicine, Kyoto University, 54 Shogoin-Kawahara-cho, Sakyo-ku, 606-8507, Kyoto, Japan; Department of Radiation Oncology, Faculty of Medicine, Kindai University, 377-2, Ohno-Higashi, Osaka-Sayama, 589-8511, Osaka, Japan; Department of Radiation Oncology, Osaka Red Cross Hospital, 5-30 Fudegasaki-cho, Tennoji-ku, 543-8555, Osaka, Japan; Department of Radiation Oncology and Image-Applied Therapy, Graduate School of Medicine, Kyoto University, 54 Shogoin-Kawahara-cho, Sakyo-ku, 606-8507, Kyoto, Japan; Department of Radiation Oncology, Kobe City Medical Center General Hospital, Minamimachi 21-1, Minatojima, Chuo-ku, Kobe 650-0047, Hyogo, Japan

**Keywords:** angiosarcoma, scalp, face, intensity-modulated radiotherapy, multicenter study, skin ulceration

## Abstract

Combined modality therapy, including radiotherapy (RT), is a common treatment for scalp or face angiosarcoma. Although intensity-modulated radiotherapy (IMRT) can deliver homogeneous doses to the scalp or face, clinical data are limited. This multicenter study aimed to evaluate scalp or face angiosarcoma treated with definitive or post-operative IMRT. We retrospectively analyzed data from patients who received IMRT for scalp or face angiosarcoma at three institutions between January 2015 and March 2020. Local control (LC) rate, overall survival (OS), progression-free survival (PFS), recurrence patterns and toxicity were evaluated. Fifteen patients underwent IMRT during the study period. Definitive RT was performed on 10 patients and post-operative RT was performed on 5 patients. The 1-year LC rate was 85.7% (95% confidence interval [CI], 53.9–96.2%). The 1-year OS and PFS rates were 66.7% (95% CI, 37.5–84.6%) and 53.3% (95% CI, 26.3%–74.4%), respectively. Univariate analysis revealed that a clinical target volume over 500 cm^3^ was associated with poor LC. Distant metastasis was the most common recurrence pattern. All patients experienced Grade 2 or 3 radiation dermatitis, and five patients experienced grade ≥ 3 skin ulceration. One patient who underwent maintenance therapy with pazopanib developed Grade 5 skin ulceration. Fisher’s exact test showed that post-operative RT was significantly associated with an increased risk of skin ulceration of grade ≥ 3. These results demonstrate that IMRT is a feasible and effective treatment for scalp or face angiosarcoma, although skin ulceration of grade ≥ 3 is a common adverse event in patients who receive post-operative RT.

## INTRODUCTION

Angiosarcoma is a rare and aggressive malignant tumor, and approximately half of angiosarcomas occur on the skin of elderly individuals. The scalp or face is the most common site for an angiosarcoma, accounting for ~50–60% of all skin angiosarcomas [1], with a poor prognosis and 5-year disease-free survival rate of <20% [2, 3].

The treatment strategy for scalp or face angiosarcoma involves combined therapy with surgical excision, chemotherapy and radiotherapy (RT) [2, 4, 5]. Although the main recurrence pattern of scalp or face angiosarcoma is distant metastasis, local disease causes bleeding, pain and cosmetic problems, leading to physical and psychological distress that reduces the patients’ quality of life [6]. Thus, local control (LC) of scalp or face angiosarcoma is important, and RT plays an important role in the treatment of angiosarcoma, especially for large lesions where surgery is difficult. However, the conventional RT technique of combining two pairs of lateral X-rays and electron fields [7–9] for scalp or face angiosarcoma is complicated, and the 1-year LC rate is ~50–70%, which is not sufficient [10, 11]. The difficulty in delivering a homogenous dose to the spherical surface of the scalp or face and the proximity to the eye or brain make it challenging to cover the target with sufficient margin. By contrast, intensity-modulated radiotherapy (IMRT) can deliver a homogenous dose to the target with a lower organ-at-risk (OAR) dose [12, 13], and volumetric modulated arc therapy (VMAT), a technique of IMRT, can irradiate the target with a short treatment delivery time. Previous studies have reported dosimetric superiority of these techniques on the scalp and face [14, 15]; however, there are limited reports of the clinical outcomes of scalp or face angiosarcoma treated with IMRT or VMAT, and all of them are single-institution reports [16, 17].

Therefore, the purpose of this multicenter study was to assess the efficacy and feasibility of IMRT in patients with scalp or face angiosarcoma.

## MATERIALS AND METHODS

### Patient selection

A retrospective multicenter study was conducted on patients with biopsy-proven angiosarcoma of the scalp or face treated with curative IMRT between January 2015 and March 2020 at three institutions (Kobe City Medical Center General Hospital, Kyoto University Hospital and Osaka Red Cross Hospital). The study included patients aged ≥20 years with a planned equivalent dose of 2 Gy fraction of a total of ≥54 Gy, regardless of whether surgery or systemic therapy was performed. Patients with a history of RT for scalp or facial lesions were excluded. Informed consent was waived, and the study was approved by the Institutional Review Board of Kobe City Medical Center General Hospital (zh230424).

### Data collection

Information regarding patient status, tumor and treatment characteristics, including surgery, RT and systemic therapy, was obtained from clinical records. Systemic therapy, which involves chemotherapy and molecular targeted therapy, was classified into induction, concurrent and maintenance systemic therapies. The biological effective dose (BED) was used in a linear-quadratic model to evaluate several total doses or fraction sizes. The BED was calculated using the formula: *nd*(1 + *d*/*α*/*β*) Gy, where ‘*n*’ is the fractionation number, ‘*d*’ is the daily dose and ‘*α*/*β*’ is assumed to be 10 for tumors.

### Evaluation and statistical analysis

The primary endpoint was the 1-year LC rate, and the secondary endpoints were overall survival (OS), progression-free survival (PFS), recurrence pattern and toxicity. The LC was calculated from the date of initiation of the first treatment (surgery, RT or systemic therapy) to the date of local recurrence, defined as recurrence in the radiation field or marginal area. OS was calculated from the date of initiation of the first treatment to the date of death by any cause and was censored at the last follow-up visit for living patients. PFS was calculated from the date of initiation of the first treatment until disease progression or death. Disease progression was defined as the evidence of progressive disease on inspection, palpation or diagnostic imaging. Recurrence patterns were categorized into local recurrence, regional recurrence (involving recurrences occurring on the scalp, face or neck skin apart from the irradiated site or cervical lymph nodes metastasis) and distant metastasis.

Statistical analyses were performed using EZR version 1.55 (Saitama Medical Center, Jichi Medical University, Saitama, Japan). Kaplan–Meier technique was used to estimate the LC, OS and PFS. The log-rank test was used for univariate analysis. Toxicities were classified as acute (from the initiation of RT to 28 days after RT) or late, and the Common Terminology Criteria for Adverse Events version 5.0 was used for the assessment of toxicities. We set a shorter evaluation period of acute toxicity to mitigate the effect of maintenance systemic therapy. Skin ulceration included graft failure in post-operative cases. The Fisher’s exact test was used to compare the incidence of grade ≥ 3 acute hematologic toxicities and skin ulceration with various factors. A *P-*value <0.05 was considered to be statistically significant using the log-rank test and Fisher’s exact test.

## RESULTS

### Patient characteristics

Fifteen patients (five from each institution) were enrolled in this study; all patients had been diagnosed with scalp or face angiosarcoma and had undergone definitive or post-operative IMRT between January 2015 and March 2020. Before treatment, all patients underwent computed tomography (CT) or ^18^F-fluorodeoxyglucose-positron emission tomography (FDG-PET) imaging. After treatment, periodic follow-up was conducted, including physical and blood examinations, to monitor for recurrence or toxicities, as well a CT scan of the chest and abdomen or FDG-PET. Except in one case, the interval of imaging examinations was 3–6 months. Patient characteristics are presented in Table 1. The median tumor diameter was 9.5 cm (range, 1.7–33.0 cm), and the primary lesion was located on the scalp in 14 patients and on the face in 1 patient. Seven patients had solitary tumors, and eight had satellite lesions. At diagnosis, two patients had muscle invasion, while none had bone invasion.

**Table 1:** Patient and tumor characteristics (*n* = 15)

Characteristics	No.	%	Median (range)
Age (years)			74 (66–90)
Sex			
Male	10	66.7	
Female	5	33.3	
Performance status			
0 1	10 5	66.7 33.3	
Tumor diameter (cm)			9.5 (Range, 1.7–33.0)
Tumor location			
Scalp Face	14 1	93.3 6.7	
Skip lesions	8	53.3	
With nodule lesions	11	73.3	
With ulcerative lesions	5	33.3	
With muscle invasion	2	13.3	
Lymph node metastasis	2	13.3	
With parotid gland invasion	1	6.7	

### Treatment

#### Surgery

Table 2 describes the variation in treatment among the 15 patients. Of the patients, five underwent surgery, with a margin of 1–2 cm from the gross lesion for four patients and without margin for one patient. Surgical excision was performed when the tumor size or general condition was acceptable. The reasons for not performing surgery in patients with definitive RT were as follows: a large lesion in four patients, the risk to critical facial structures (e.g. lateral canthus) in two patients and unclear data in the clinical records of four patients. A skin graft was used for all surgical excisions. Lateral and vertical margins were positive in four and three patients, respectively.

**Table 2:** Treatment details

Surgery	No.	%	Median (range)
Performed surgery			
Yes No	5 10	33.3 66.7	
Surgical margin			
Positive	4	80	
Negative	1	20	
RT			
Administered dose (BED)			
70 Gy in 35 fractions (84) 66 Gy in 30 fractions (80.5) 72 Gy in 30 fractions (89.3) 52.9 Gy in 23 fractions + 15 Gy in 5 fractions (84.6) 67.2 Gy in 28 fractions (83.3) 60 Gy in 20 fractions (78) 60 Gy in 30 fractions (72)^a^ 50 Gy in 25 fractions (60)^a^	7 2 1 1 1 1 1 1	46.7 13.3 6.7 6.7 6.7 6.7 6.7 6.7	
Boost technique			
SIB technique SEQ technique Both No	7 1 2 5	46.7 6.7 13.3 33.3	
Total scalp irradiation			
Yes No	11 4	73.3 26.7	
Lymph node irradiation^b^			
Yes No	3 12	20 80	
CTV (cm^3^)			473.8 (59.8–1657.9)
Systemic therapy			
Induction systemic therapy			
Weekly paclitaxel Monthly paclitaxel No	7 2 6	46.7 13.3 40	
Concurrent systemic therapy			
Weekly paclitaxel Monthly paclitaxel Weekly docetaxel Pazopanib No	10 2 1 1 1	66.7 13.3 6.7 6.7 6.7	
Maintenance systemic therapy			
Weekly paclitaxel Monthly paclitaxel Monthly docetaxel Pazopanib No	4 5 1 2 3	26.7 33.3 6.7 13.3 20	

Abbreviations: SIB = simultaneous integrated boost, SEQ = sequential boost.

^a^Dropped out before completing RT.

^b^Irradiation administered to metastatic lymph node, irradiation administered to prophylactic lymph node or both.

#### Radiotherapy

All patients underwent a 2-mm or 2.5-mm slice simulation CT, and a head shell was used to stabilize patients with a 5–10-mm bolus to ensure sufficient irradiation of the epidermis. The gross tumor volume (GTV) consisted of the primary or satellite tumors and metastatic lymph nodes. The GTV was determined by placing metal wires on the planning CT, and in some cases, a dermatologist was involved in the planning process to mark the GTV. The clinical target volume (CTV) included a margin of ≥3 cm from the tumor in all definitive RT cases or a margin of ≥3 cm from the surgical edge in all post-operative cases. The depth-wise extent of the CTV was up to the muscle for the head and up to the subcutaneous tissue for the facial region and posterior neck. In 11 patients, the total scalp was irradiated due to the presence of a large lesion and/or a wide CTV margin. Additionally, prophylactic lymph nodes were included in two patients, and the salivary gland was included in one patient. The planning target volume (PTV) was the CTV with a margin of 3–5 mm in all directions. The extent of the PTV varied among the patients regardless of whether it encompassed the surrounding bolus or not; nevertheless, a sufficient dose was administered to the skin in PTV in all cases. Planning was performed using the commercially available planning system Eclipse™ (Varian Medical Systems, Palo Alto, CA, USA).

All patients were treated with VMAT with two to four arcs, four to six MV X-rays, and one patient was treated with VMAT and 6 MeV electron boost. The major dose and fractions were 70 Gy in 35 fractions (BED, 84) for 7 patients, and the median BED was 84 (range, 60–89.3). The prescribed prophylactic doses to the cervical lymph node were 54 Gy in 30 fractions and 50 Gy in 25 fractions. The total dose was similar between definitive and post-operative RT because the lesions were considered to remain due to reasons such as positive resection margins in post-operative RT cases. Table 3 lists the dose constraints, and Supplementary Table 1 presents the dose volume index of other organs at risk on planning CT. The prescription dose was delivered as the dose covering 50% of the PTV. To irradiate the prophylactic area with a lower dose, the simultaneous integrated boost technique was adopted for seven patients, the sequential boost technique was adopted for one patient and both techniques were adopted for two patients. The median overall treatment period for RT was 49 days (range, 30–61 days). Two patients required a modified RT plan due to radiation dermatitis for one and tumor progression and radiation dermatitis for the other. In four patients, RT was suspended due to Grade 3 dermatitis (*n* = 3) or Grade 4 neutropenia (*n* = 1). Two patients dropped out of RT at 50 and 60 Gy owing to the incidence of non-inclusive mesenteric ischemia and anorexia, respectively.

**Table 3:** Dose constraints

Structure	Constraints
Spinal cord	Dmax < 45 Gy
Brainstem	Dmax < 54 Gy
Optic nerves	Dmax < 54 Gy

Abbreviations: Dmax = maximum dose to the structure volume.

#### Systemic therapy

Nine patients underwent induction systemic therapy, 14 underwent concurrent systemic therapy and 12 underwent maintenance systemic therapy. Paclitaxel was the most frequently used agent for systemic therapy, with a typical dose of 80 mg/m^2^ per week and 175 mg/m^2^ per month. If disease progression was detected, and the patient’s condition allowed it, second-line systemic therapy was initiated.

### Treatment outcomes and recurrence pattern

**Fig. 1:**
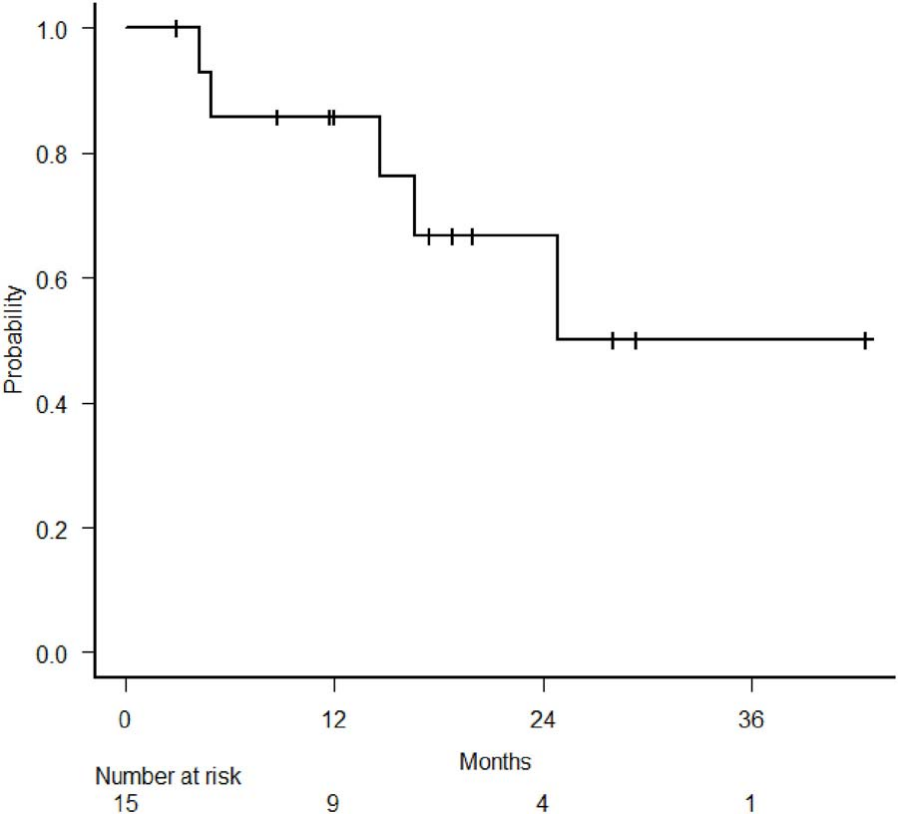
Kaplan–Meier estimates of LC.

**Fig. 2:**
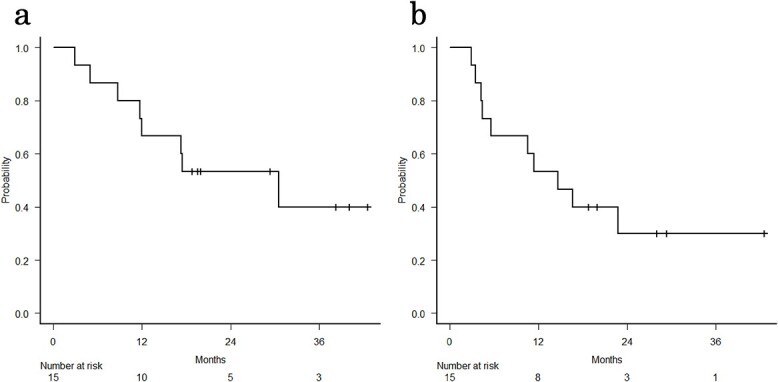
Kaplan–Meier estimates of (**a**) OS and (**b**) PFS.

Out of the 15 patients analyzed, 7 (46.7%) were alive at the time of the analysis. The median follow-up period for all patients was 18.8 months (range, 3.0–42.6 months), while for survivors, it was 29.4 months (range, 18.8–42.6 months). There was one alive patient whose follow-up period was the same as the median follow-up period for all the patients. The 1-year LC rate was 85.7% (95% confidence interval [CI], 53.9–96.2%; Fig. 1). The median survival time (MST) was 30.6 months (95% CI, 8.8–NA), the median PFS was 14.6 months (95% CI, 4.3–NA) and the 1-year OS and PFS rates were 66.7% (95% CI, 37.5–84.6%) and 53.3% (95% CI, 26.3%–74.4%), respectively (Fig. 2). In the definitive RT group, the 1-year LC, OS and PFS rates were 88.9% (95% CI, 43.3–98.4%), 70.0% (95% CI, 32.9–89.2%) and 60.0% (95% CI, 25.3–82.7%), respectively. In the post-operative RT group, the 1-year LC, OS and PFS rates were 80.0% (95% CI, 20.4–96.9%), 60.0% (95% CI, 12.6–88.2%) and 40.0% (95% CI, 5.2–75.3%), respectively.

During the follow-up period, recurrence was observed in nine patients (60%): six patients in the definitive RT group and three patients in the post-operative RT group (Table 4). Local recurrence was observed in five patients, while regional recurrence was observed in two patients: in the cervical lymph node in one case and on the skin of the neck in another. Notably, there was no recurrence on the scalp outside the irradiation field among the patients who did not receive total scalp irradiation. Lung metastasis was the most common distant metastasis, occurring in six patients. The cause of death was tumor progression in six patients, non-occlusive mesenteric ischemia in one patient and treatment-related complications in one patient. The univariate analysis showed that a CTV of over 500 cm^3^ was statistically related to poor LC (Table 5). The presence of large lesion, skip lesions, nodule and muscle invasion tended to have poor LC, but the correlation was not statistically significant. High BED and surgery were not prognostic factors in this analysis.

**Table 4:** Recurrence pattern and treatment characteristics

No.	Age (years)	Sex	PS	Tumor diameter (cm)	Site	With nodule	With ulcer	Skip lesion	Surgery	Administered RT dose	Total scalp irradiation	Systemic therapy	Months to disease recurrent (site)
1	66	M	0	4.5	Scalp	−	+	+	+	70 Gy/35 fr	+	I + C + M	5 (R + D)
2	71	M	1	14	Scalp	+	−	−	−	66 Gy/30 fr	+	I + M	23 (D), 25 (L + R)
3	74	M	0	6	Scalp	−	−	−	−	67.2 Gy/28 fr	−	C + M	4 (D)
4	70	M	0	18	Scalp	+	−	+	−	72 Gy/30 fr	+	I + C + M	10 (D)
5	73	M	0	18	Scalp	+	+	+	+	66 Gy/30 fr	+	I + C + M	11 (D)
6	66	M	0	10	Scalp	+	−	+	+	70 Gy/35 fr	+	I + C + M	4 (L)
7	90	F	1	30	Scalp	+	+	+	−	60 Gy/30 fr	+	I + C	3 (D), 5 (L)
8	78	M	0	33	Scalp	+	−	−	−	70 Gy/35 fr	+	C + M	14 (L)
9	83	M	1	7	Scalp	+	−	+	−	70 Gy/35 fr	+	C + M	16 (L + D)

Abbreviations: PS = performance status, I = induction systemic therapy, C = concurrent systemic therapy, M = maintenance systemic therapy, R = regional recurrence, D = distant metastasis, L = local recurrence.

**Table 5:** Univariate analysis of the potential prognostic factors for LC

Variables	No. (*n* = 15)	1-year LC rate (%), (95% CI (%))	*P*-value
Sex			0.28
Male	10 (66.7%)	88.9 (43.3–98.4)	
Female	5 (33.3%)	80.0 (20.4–96.9)	
Age (years)			0.26
<74	9 (60%)	88.9 (43.3–98.4)	
≥75	6 (40%)	80.0 (20.4–96.9)	
Performance status			0.26
0	10 (66.7%)	90.0 (47.3–98.5)	
1	5 (33.3%)	75.0 (12.8–96.1)	
Tumor diameter			0.069
<10 cm	8 (53.3%)	100 (NA-NA)	
≥10 cm	7 (46.7%)	71.4 (25.8–92.0)	
Skip lesions			0.091
Yes	8 (53.3%)	71.4 (25.8–92.0)	
No	7 (46.7%)	NA	
With nodule lesions			0.13
Yes	11 (73.3%)	80.0 (40.9–94.6)	
No	4 (26.7%)	100 (NA-NA)	
With ulcerative lesions			0.93
Yes	5 (33.3%)	75.0 (12.8–96.1)	
No	10 (66.7%)	90.0 (47.3–98.5)	
With muscle invasion			0.076
Yes	2 (13.3%)	NA	
No	13 (86.7%)	91.7 (53.9–98.8)	
Surgery			0.70
Yes	5 (33.3%)	80.0 (20.4–96.9)	
No	10 (66.7%)	88.9 (43.3–98.4)	
BED ≥84			0.96
Yes	9 (60%)	88.9 (43.3–98.4)	
No	6 (40%)	80.0 (20.4–96.9)	
CTV ≥ 500 cm^3^			**0.0012**
Yes	7	66.7 (19.5–90.4)	
No	8	1.00 (NA-NA)	

Abbreviations: NA = not applicable.

### Toxicities

Table 6 shows data on acute and late adverse events in all patients. Among the acute hematologic toxicities of Grade 3 or higher, anemia, neutropenia and thrombocytopenia were observed in one patient, three patients and one patient, respectively. The incidence of Grade 3 or higher acute hematologic toxicities was not associated with the use of induction or concurrent systemic therapy (Supplementary Table 2). Regarding acute non-hematologic toxicity, Grade 2 and 3 dermatitis occurred in 3 and 12 patients, respectively, and Grade 2 and 3 mucositis occurred in 4 and 1 patients, respectively. Late toxicity Grade 2, 3, and 5 skin ulcerations were observed in two, four, and one patient, respectively. In patients with Grade 3 or 5 skin ulceration, four out of five patients underwent post-operative RT, and the patient with Grade 5 skin ulceration died at 506 days after starting RT owing to a *Pseudomonas aeruginosa* infection from ulceration. This patient underwent surgery and received concurrent post-operative chemoradiotherapy at a dose of 70 Gy in 35 fractions with weekly paclitaxel, followed by maintenance therapy with pazopanib at a dose of 200 mg/day from the day after completion of RT. Pazopanib was temporarily paused for 3 weeks due to thrombocytopenia. While exacerbation of dermatitis was found upon resumption, there was no persistent worsening, leading to its continued administration of pazopanib at a final dose of 400 mg/day. The patient experienced local recurrence 4 months after the start of treatment, apart from the surgical site. The patient continued treatment with pazopanib, and on the FDG-PET scan after 4 months from recurrence, the recurrent lesion showed complete response (CR). Four months before death, expansion of the ulcer at the surgical site was observed, although the recurrent lesion still maintained CR. However, the ulcer at the surgical site was still expanding, and the presence of *P. aeruginosa* was detected. Maintenance pazopanib was administered until 1 month before death.

**Table 6:** Toxicities of Grade 2 or higher

Toxicity	Grade 2	Grade 3	Grade 4	Grade 5
Acute toxicities				
Anemia	5	1	0	0
Neutropenia	2	1	2	0
Thrombocytopenia	0	0	1	0
Radiation dermatitis	3	12	0	0
Mucositis	4	1	0	0
Dysgeusia	4	0	0	0
Hyponatremia	0	1	0	0
Anorexia	1	0	0	0
Constipation	1	0	0	0
Late toxicities				
Skin ulceration	2	4	0	1
Peripheral sensory neuropathy	1	0	0	0

No clinical or radiological central nervous system necrosis, otitis externa or corneal ulcerations were observed during the observation period. The Fisher’s exact test indicated that the incidence of Grade 3 or higher skin ulceration was significantly higher in the post-operative RT group than that in the definitive RT group (*P* = 0.017). The incidence of Grade 3 acute radiation dermatitis was not associated with the incidence of grade ≥ 3 skin ulceration. The results of the Fisher’s exact test are described in Table 7.

**Table 7:** Fisher’s exact test for grade 3 or higher skin ulceration

Variables	Grade ≥ 3 skin ulceration (*n* = 5)	Grade < 3 skin ulceration (*n* = 10)	*P*-value
Sex			0.60
Male	4	6	
Female	1	4	
Age (years)			0.58
<74	4	5	
≥75	1	5	
Performance status			0.10
0	5	5	
1	0	5	
Tumor diameter ≥ 10 cm	3	4	0.61
With nodule lesions	4	7	1
With ulcerative lesions	2	3	1
With muscle invasion	2	0	0.095
Post-operative RT	4	1	**0.017**
BED ≥84	4	5	0.58
CTV ≥ 500 cm^3^	2	5	1
With induction systemic therapy	4	5	0.58
With concurrent systemic therapy	5	9	1
With maintenance systemic therapy	4	8	1
Grade 3 radiation dermatitis	5	7	0.51

## DISCUSSION

This multicenter study investigated the efficacy and feasibility of IMRT for scalp or face angiosarcoma, which, to the best of our knowledge, is the first multicenter study on this topic. Angiosarcoma often requires a large irradiation field, owing to its tendency of involving wider invasion than is initially apparent; however, delivering a homogenous dose to the target was difficult with the conventional RT technique, owing to the spherical shape of the scalp. IMRT has the advantage of being able to deliver an accurate dose to the target, which has the potential to improve outcomes, particularly in LC. Previous reports on the treatment of angiosarcoma with conventional X-ray and electron beam have reported a 1-year LC and MST of 57–71 and 8–31 months, respectively [8, 11, 18, 19]. Compared to these past results, our study showed favorable survival outcomes and at least non-inferior LC. Kashihara *et al*. reported a 2-year local progression-free rate of 48.4% and an MST of 9 months for scalp or face angiosarcoma treated with definitive IMRT [17]. Our 1-year LC and OS rates in the definitive RT group were 88.9 and 70.0%, respectively, which were considered to be non-inferior to these results.

Univariate analysis revealed that a larger CTV was a poor prognostic factor for LC, and a large CTV was found to be a poor prognostic factor for the local progression-free rate in a previous report [17]. A large CTV was associated with high tumor diameter or skip lesion, which may have resulted in worse LC. By contrast, high BED, which was reported as a good prognostic factor in other studies, was not statistically significant in our analysis [11, 18]. One possible reason is the superiority of the dose distribution of IMRT. Suzuki *et al*. reported that a total BED of ≥95 Gy is associated with improved LC among scalp angiosarcoma patients treated with conventional RT and electrons [11]. The BED used in our study was mainly 84 Gy, and the maximum BED did not reach 95 Gy; nevertheless, the LC rate was satisfactory. Kashihara *et al*. and Okano *et al*. also reported good results with IMRT for scalp angiosarcoma, with a 2-year local progression-free rate of 48.4% and a 1-year LC rate of 100%, respectively, with a median BED of 84 Gy, which was the same as ours [17, 20]. IMRT can deliver a more homogeneous dose to the target, even in cases with extensive lesions or those in close proximity to OAR, which may contribute to LC without the need for dose escalation.

As shown in Table 6, Grade 2 or 3 dermatitis occurred in all the patients during RT. Radiation dermatitis is almost certain to occur in cases of RT to scalp or face angiosarcoma, as noted in other studies [9, 17, 19]. Therefore, further dose escalation with IMRT should be approached with caution because LC was observed to be good in our study, as mentioned above. Additionally, considering that four patients in our study needed to pause RT due to dermatitis, the management of dermatitis is important. Radiation dermatitis commonly occurs with RT in the breast, head and neck regions, and the benefits of washing or preventive topical steroids have been reported in these regions [21–24]. Although the intervention of steroids on tumors or tumor beds may be difficult, skincare, including washing and topical steroids, may reduce dermatitis in the marginal region and result in the completion of RT.

In conventional RT, there is the uncertainty of overlapping irradiation fields, which may lead to higher risk of late toxicities like ulceration. We considered that IMRT may reduce late toxicities with its certainty; however, compared to results reported previously, skin ulceration was a frequent occurrence in our study, with one death occurring, owing to infection resulting from skin ulceration [9, 17]. Fisher’s exact test also revealed that post-operative RT was a significant risk factor for Grade 3 or higher skin ulceration. Although, high-dose RT (BED, 72–94.5 Gy) is usually administered in post-operative cases of scalp or face angiosarcoma, the reports of post-operative RT and skin ulceration are limited [11]. In our study, the BED ranged from 60 to 89.3 Gy, which was not extremely high. Nonetheless, caution should be exercised when administering high-dose IMRT in post-operative cases because delivering a homogenous, strong dose to the target may be a double-edged sword, which could result in stronger irradiation of the skin or skin graft than the electrons even if the prescribed dose is the same, possibly leading to a higher risk of ulceration or graft failure [14, 17, 25]. By contrast, only one patient in our study developed Grade 3 skin ulceration with definitive RT. Thus, high-dose definitive IMRT may be acceptable from the point of view of skin ulceration, given that local recurrence can cause a variety of symptoms.

Additionally, one patient had Grade 5 skin ulceration. This patient underwent maintenance systemic therapy with pazopanib, which might have caused skin ulceration as well as high-dose post-operative IMRT. Pazopanib is multireceptor tyrosine kinase inhibitors (TKI) targeting agent, including vascular endothelial growth factor, and is known for its effectiveness for angiosarcoma [26]. On the other hand, radiation recall and severe gastrointestinal toxicities were reported with multireceptor TKIs targeting agent combined with RT [27–29]. In this patient, pazopanib was administered for ~1 year after the completion of RT, and it might have hindered ulcer healing, potentially leading to Grade 5. While pazopanib is one of the few systemic therapies effective for angiosarcoma, caution should be exercised when combining it with RT.

However, with the exception of dermatitis and skin ulceration, few patients in our study experienced Grade 3 or higher toxicities. Furthermore, there were no instances of otitis externa or corneal ulcers despite the target and OAR being anatomically close, which could be attributed to IMRT. A previous study indicated that IMRT provides a higher dose to the brain than the electrons, yet there were no patients in that study with brain necrosis [14]. Moreover, considering the aggressive progression of angiosarcoma, toxicity was likely acceptable.

Total scalp irradiation has been used commonly to treat scalp angiosarcoma; however, recent studies have preferred local irradiation [17, 20]. As mentioned earlier, RT for scalp or face angiosarcoma can be challenging, and if local irradiation is feasible, it could help minimize the adverse effects. In this study, 11 out of 15 patients received total scalp irradiation due to the presence of a large lesion or sufficient CTV margin; the remaining 4 patients who received local irradiation showed no recurrence outside the irradiation field. Although our sample size was limited and conclusive statements cannot be made, our data highlight the possibility of total scalp irradiation not being universally necessary. Further analyses with larger cohorts are needed in this regard.

Our study had several limitations that should be considered when interpreting the results. First, the study cohort was small owing to the rarity of scalp or face angiosarcoma, and the study was retrospective in design, possibly causing statistical bias and uncertainty. However, despite the small cohort, we conducted a multicenter study to minimize the bias, which is considered to better reflect clinical practice than a single-center study. Another limitation was that our cohort encompassed both definitive and post-operative RT cases, so the tumor characteristics may have differed in our sample, such as tumor size or lymph node metastasis. Additionally, the study was a single-arm analysis, and the comparison between the treatment results of IMRT and conventional RT was not assessed. Finally, the toxicities were not evaluated rigorously due to the retrospective nature of the study. However, severe toxicities that required treatment were clearly recorded, while low-grade toxicities were possibly underestimated. Despite these limitations, the survival outcomes were favorable, and IMRT had various merits, including irradiation homogeneity of the target, simultaneous irradiation even when the target was large and easy matching of adjacent electron fields. In addition, VMAT can reduce the treatment time. However, high-dose post-operative IMRT should be carefully performed due to the higher risk of skin ulceration. Further investigations with a larger number of patients and comparison to other treatment modalities for scalp or face angiosarcoma patients are required to pursue the ideal treatment.

## CONCLUSION

In conclusion, IMRT showed favorable LC and survival outcomes in patients with scalp or face angiosarcoma. However, Grade 3 or higher skin ulceration commonly occurs in patients who underwent post-operative RT compared with those who underwent definitive RT, and thus, high-dose post-operative IMRT should be carefully performed.

## Supplementary Material

Supplementary_Table_1_2nd_rivise_no_highlight_rrad089

Supplementary_Table_2_no_highlight_rrad089

## PRESENTATION AT A CONFERENCE

Parts of this paper were presented at JASTRO 2021 (12–14 November 2021, Japan) and ESTRO 2023 (12–16 May 2023, Vienna, Austria).
