# Supplementary material for: Clinical outcomes of scalp or face angiosarcoma treatment with intensity-modulated radiotherapy: a multicenter study
Source: J Radiat Res. 2023 Nov 22;65(1):78–86. doi: 10.1093/jrr/rrad089 (PMC10803163; doi:10.1093/jrr/rrad089)
Supplement: Supplementary_Table_1_2nd_rivise_no_highlight_rrad089 [file supplementary_table_1_2nd_rivise_no_highlight_rrad089.docx]

**Supplementary Table 1. Dose volume index of organs at risk on the planning CT**

| **Structure** | **Index** | **Median (range)** |
| --- | --- | --- |
| Brain | Dmax | 63.6 Gy (47.2-71.6 Gy) |
|  | Dmean | 26.1 Gy (6.1-32.0 Gy) |
| Eyes | Dmax | 36.4 Gy (9.0-51.9 Gy) |
| Retinas | Dmax | 37.1 Gy (9.0- 47.6 Gy) |
| Lenses | Dmax | 14.9 Gy (6.9-35.7 Gy) |
| Cochleas | Dmean | 18.6 Gy (2.6-31.5 Gy) |
| Right lacrimal gland | Dmax | 30.9 Gy (8.1-52.3 Gy) |
|  | Dmean | 23.4 Gy (5.0-50.3 Gy) |
| Left lacrimal gland | Dmax | 25.6 Gy (7.4-41.4 Gy) |
|  | Dmean | 17.6 Gy (4.8-34.9 Gy) |
| Parotid gland (spared) | Dmean | 13.6 Gy (0.7-47.4 Gy) |
|  | V30 Gy | 0.4% (0-100%) |
| Parotid glands | Dmean | 15.4 Gy (0.7-48.2 Gy) |
|  | V30 Gy | 12.8% (0-100%) |

Abbreviations: Dmax, the maximum dose to the structure volume; Dmean, the mean dose to the structure volume; V30 Gy, the volume of the structure receiving ≥ 30 Gy
