# Supplementary material for: Clinical outcomes of scalp or face angiosarcoma treatment with intensity-modulated radiotherapy: a multicenter study
Source: J Radiat Res. 2023 Nov 22;65(1):78–86. doi: 10.1093/jrr/rrad089 (PMC10803163; doi:10.1093/jrr/rrad089)
Supplement: Supplementary_Table_2_no_highlight_rrad089 [file supplementary_table_2_no_highlight_rrad089.docx]

**Supplementary Table 2. Fisher’s exact test for grade 3 or higher hematologic toxicities**

| Variables | Grade ≥ 3 hematologic toxicities (n=3) | Grade < 3 hematologic toxicities (n=12) | *p*-value |
| --- | --- | --- | --- |
| Sex |  |  | 0.51 |
| Male | 3 | 7 |  |
| Female | 0 | 5 |  |
| Age (years) |  |  | 0.53 |
| < 74 | 1 | 8 |  |
| ≥ 75 | 2 | 4 |  |
| Performance status |  |  | 0.24 |
| 0 | 1 | 9 |  |
| 1 | 2 | 3 |  |
| Tumor diameter ≥ 10 cm | 1 | 6 | 1 |
| Postoperative radiotherapy | 1 | 4 | 1 |
| BED ≥ 84 | 2 | 7 | 1 |
| CTV ≥ 500 cm^3^ | 3 | 4 | 0.077 |
| With induction systemic therapy | 2 | 7 | 1 |
| With concurrent systemic therapy | 3 | 11 | 1 |

Abbreviations: BED, biological effective dose; CTV, clinical target volume
